# In-depth resistome analysis by targeted metagenomics

**DOI:** 10.1186/s40168-017-0387-y

**Published:** 2018-01-15

**Authors:** Val F. Lanza, Fernando Baquero, José Luís Martínez, Ricardo Ramos-Ruíz, Bruno González-Zorn, Antoine Andremont, Antonio Sánchez-Valenzuela, Stanislav Dusko Ehrlich, Sean Kennedy, Etienne Ruppé, Willem van Schaik, Rob J. Willems, Fernando de la Cruz, Teresa M. Coque

**Affiliations:** 1 Department of Microbiology, Ramón y Cajal University Hospital, Ramón y Cajal Health Research Institute (IRYCIS), Madrid, Spain; 2 Joint Unit of Antibiotic Resistance and Bacterial Virulence associated with the Spanish National Research Council (CSIC), Madrid, Spain; 3 Network Research Center for Epidemiology and Public Health (CIBER-ESP), Madrid, Spain; 4 National Center of Biotechnology, CSIC, Madrid, Spain; 5 Genomics Unit, Madrid Science Park, Madrid, Spain; 6 Faculty of Veterinary Medicine, Complutense University of Madrid, Madrid, Spain; 7 IAME, UMR 1137, INSERM, Paris Diderot University, Sorbonne Paris Cité, Bacteriology Laboratory, Hospital Bichat, AP-HP, Paris, France; 8 MGP MetaGénoPolis, INRA, University of Paris-Saclay, Jouy-en-Josas, France; 9 Center of Host Microbiome Interactions, King’s College, London, UK; 10 Department of Medical Microbiology, University Medical Center, Utrecht, Netherlands; 11 Department of Molecular Biology, University of Cantabria, Santander, Spain; 12 Institute of Biomedicine and Biotechnology of Cantabria, IBBTEC (UC-CSIC), Santander, Spain; 13 Present Address: Bioinformatics and Biostatistics HUB, C3BI and Biomics Pole, CITECH Pasteur Institute, Centre François Jacob, Paris, France; 14 Present Address: Institute of Microbiology and Infection, University of Birmingham, Birmingham, B15 2TT UK

**Keywords:** Antimicrobial resistance, Resistome, Metagenomics, Differential abundance analysis, Targeted metagenomics

## Abstract

**Background:**

Antimicrobial resistance is a major global health challenge. Metagenomics allows analyzing the presence and dynamics of “resistomes” (the ensemble of genes encoding antimicrobial resistance in a given microbiome) in disparate microbial ecosystems. However, the low sensitivity and specificity of available metagenomic methods preclude the detection of minority populations (often present below their detection threshold) and/or the identification of allelic variants that differ in the resulting phenotype. Here, we describe a novel strategy that combines targeted metagenomics using last generation in-solution capture platforms, with novel bioinformatics tools to establish a standardized framework that allows both quantitative and qualitative analyses of resistomes.

**Methods:**

We developed ResCap, a targeted sequence capture platform based on SeqCapEZ (NimbleGene) technology, which includes probes for 8667 canonical resistance genes (7963 antibiotic resistance genes and 704 genes conferring resistance to metals or biocides), and 2517 relaxase genes (plasmid markers) and 78,600 genes homologous to the previous identified targets (47,806 for antibiotics and 30,794 for biocides or metals). Its performance was compared with metagenomic shotgun sequencing (MSS) for 17 fecal samples (9 humans, 8 swine). ResCap significantly improves MSS to detect “gene abundance” (from 2.0 to 83.2%) and “gene diversity” (26 versus 14.9 genes unequivocally detected per sample per million of reads; the number of reads unequivocally mapped increasing up to 300-fold by using ResCap), which were calculated using novel bioinformatic tools. ResCap also facilitated the analysis of novel genes potentially involved in the resistance to antibiotics, metals, biocides, or any combination thereof.

**Conclusions:**

ResCap, the first targeted sequence capture, specifically developed to analyze resistomes, greatly enhances the sensitivity and specificity of available metagenomic methods and offers the possibility to analyze genes related to the selection and transfer of antimicrobial resistance (biocides, heavy metals, plasmids). The model opens the possibility to study other complex microbial systems in which minority populations play a relevant role.

**Electronic supplementary material:**

The online version of this article (10.1186/s40168-017-0387-y) contains supplementary material, which is available to authorized users.

## Background

Antimicrobial resistance is considered a major global health challenge that has been recently included in the agendas of major international bodies [1]. The adoption of measures to address the antibiotic resistance crisis [2] is impaired by the controversy over what resistance is and how and where it should be detected and analyzed [3–5].

Metagenomic methods are increasingly being used to analyze the ensemble of genes that may encode antibiotic resistance in various microbial ecosystems, which are defined as the *resistome* [6–17]. An important hurdle confronting current resistome analyses is low sensitivity in the detection of minority populations harboring resistance genes (often present at concentrations below the detection level of the methods used) [18] and/or low specificity in the identification of allelic variants that might confer different susceptibility phenotypes [19]. Such requirements are needed for prioritizing risks of antibiotic resistance genes in metagenomes in terms of public health [20, 21].

The challenge of achieving a sensitive and specific identification of genes influencing antibiotic resistance in a complex metagenome background parallels the difficulties found by scientists studying human inherited diseases years ago [20]. In that case, the limitations of available sequencing technologies were overcome by using capture-based or targeted sequencing strategies of all protein-coding regions (exome). Such approaches reduced the number of sequences to be screened, and therefore represented a cost- and time-effective and high-throughput alternative to the metagenomic technologies for analyzing exomes [22, 23]. In-solution targeted capture platforms (TCPs) also provide technical improvements over array-based platforms and other genome-partitioning approaches in terms of scalability, cost-effectiveness, and enhanced data quality, including lower variance in target coverage, more accurate single nucleotide polymorphism [SNP] calling, higher reproducibility and better quality assembly [24]. Although TCPs are mostly used for the diagnosis of human inherited diseases [25], the methodology offers tremendous potential for boosting advances in environmental and ecological studies which requires the isolation of sequences of interest from a mixture of DNA from a complex community of organisms [26].

This study reports the development and validation of the first TCP for the analysis of bacterial resistomes, which was designated ResCap (for Resistome Capture). We show that ResCap could significantly improve the sensitivity and specificity over previous metagenomic analysis in the detection of antibiotic resistance. ResCap also allows the analysis of the presence and diversity of genes conferring resistance to other antimicrobials (heavy metals and biocides), which are frequently co-selected with antibiotic resistance genes and genes from replicons of mobile genetic elements (such as plasmids). An ad hoc advanced bioinformatics pipeline, developed in parallel, exploits the capabilities of ResCap for comparative metagenomic analysis. The metagenomic approach described here paves the way for a future series of applications in the studies directed to the identification, epidemiological surveillance, ecology, and study of evolutionary trajectories of resistance genes in complex microbial environments.

## Methods

### ResCap design

The ResCap capture library is a homemade core reference database (which will be available upon request) that comprises both well-known and hypothetical genes encoding resistance to antimicrobials (antibiotics, heavy metals, biocides) and genes coding for relaxases, enzymes involved in the process of DNA mobilization and transfer of conjugative elements that use to carry antimicrobial resistance genes (plasmids, conjugative transposons). The core reference database was built by downloading sequences associated with non-redundant antimicrobial genes available in the curated databases Arg-ANNOT [27], CARD [28], RED-DB (http://www.fibim.unisi.it/REDDB/Default.asp), ResFinder [29], and Bacmet [30]. These antibiotic resistance databases were combined within a non-redundant set. Proteins were clustered in protein families by homology, using CD-HIT with parameters of 80% identity and 80% coverage. Each protein family was aligned by MUSCLE v. 3.7 [31] with default parameters and a hidden Markov model (HMM) was built for each family with *hmmbuild* function of the HMMER3 [32] using default parameters. Hmmer search function (hmmsearch) was used against UniProtDB for each HMM profile to search homologous proteins for each family of proteins that confer antibiotic resistance. Manual curation of the database, which consisted on reviewing the annotation and the score of the search of datasets, allowed removing false positives. The proteins of the final data set were translated to a DNA sequences using ENA accession numbers associated with each UniProtDB entry.

The final ResCap-targeted sequence panel consists of 78,600 non-redundant genes (81,117 redundant genes) with a target space of 88.13 Mb, not yet reaching the 200 Mb target capacity offered by the custom SeqCap EZ library format (NimbleGen, Madison, USA). Probes targeting the antibiotic resistome include 47,806 putative antibiotic resistance genes and 7963 functionally characterized genes which are designed here as “canonical, antibiotic resistance genes.” Probes targeting the metal and biocide resistome include 30,794 putative resistance genes and 704 canonical resistance genes. The platform also includes probes for 2517 relaxases genes of the ConjDB database [33], which are used for plasmid identification and classification) [34]. In addition to the 8667 genes that confer functionally proven resistance to these antimicrobials (canonical genes), the platform also includes targets for 78,600 resistance gene homologs (47,806 for antibiotic and 30,794 for biocide and metal resistance).

We submitted the consolidated list of target sequences to Roche NimbleGen (Madison, USA) for capture, library design, and synthesis, which was further implemented under the custom NimbleGen SeqCap EZ Developer Library format. Redistribution of probes for better capture uniformity, redundancy and comprehensive target base coverage relied on NimbleGen based on patented algorithms. ResCap design covers 98.3% of the 88.13 Mb, and 99.6% of the genes have more than 50% of their sequence covered [Additional file 1].

### The ResCap workflow

The ResCap workflow consists of (i) whole-metagenome shotgun library construction; (ii) hybridization and capture, (iii) captured DNA sequencing. All the steps were performed according to NimbleGen standard protocols for Illumina platforms. To evaluate ResCap efficiency, the samples were sequenced before and after capture.

i) Whole-metagenome shotgun library construction. Total nucleic acid was extracted following the standardized Metahit protocol [35] (http://www.metahit.eu/) and using the FastPrep instrument (MP Biomedicals, USA). Libraries were prepared following the instructions of the Kapa Library Preparation Kit for Illumina platforms (Kapabiosystems, KR0935-v1.13). Briefly, 1.0 μg input DNA (measure by Picogreen) was fragmented to 500–600 bp insert size by sonication with Bioruptor (FastPrep®-24). After end repair, A-tailing, and adapter ligation, we followed Dual-SPRI size selection, adding 0.5 vol in the first cut and 0.2 vol in the second cut to achieve 650–750 bp libraries. Library amplification was performed using an LM-PCR of 7 cycles, as indicated in the SeqCap EZ Library SR User’s Guide v4.2. At this level, samples were labeled with specific barcodes for further sample identification. A first aliquot of the resulting amplified libraries was quality checked on a Bioanalyzer 2100 (Agilent) and pooled in equimolecular amounts for sequencing on an Illumina HiSeq 2000 instrument, generating 100–150 bp paired-end reads (pre-capture samples).

ii) Hybridization and capture. The second part of each DNA library was subjected to targeted sequence capture with the custom ResCap probes prior to sequencing (post-capture samples). Both experiments were made in separate sequencing runs. Targeted sequence capture was performed according to the manufacturer’s specifications. The captured DNA was checked for quality and integrity on a Bioanalyzer and titrated by quantitative polymerase chain reaction using the Kapa-SYBR FAST qPCR kit for LightCycler480 and a reference standard for quantification.

iii) Captured DNA sequencing. The captured libraries were denatured prior to being loaded on a flow-cell at a density of 2.2 pM, where clusters were formed and sequenced using a HiSeq 2000 in a 2 × 100 paired-end mode for swine samples and NextSeq 500 in a 2 × 150 paired-end mode for human samples. Raw sequences were processed using the FastX Toolkit (http://hannonlab.cshl.edu/fastx_toolkit/) with a quality cutoff of 20 and reads shorter than 100 and 150 bp, respectively, being discarded.

### Bioinformatic analysis

#### Reference-based workflow

**Fig. 1:**
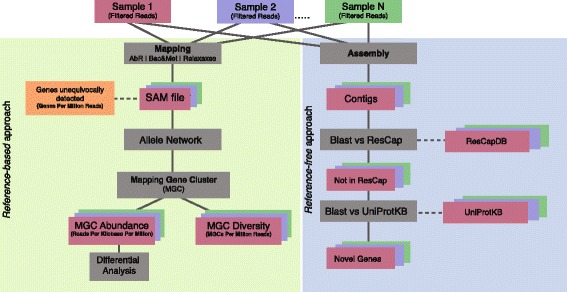
ResCap analysis workflow. Processed reads are mapped against the reference database. SAM files are parsed to extract the reads unequivocally mapped and those ambiguously mapped to determine the genes unequivocally detected and to form the allele network. The allele network is built using all the study’s SAM files. The MGCs determined from the allele network were used to perform the statistical analysis of abundance and diversity. Finally, a differential analysis was performed with the abundance data

Analysis of sequence data from metagenomes constitutes a challenge because of the inherent variability of the samples analyzed, and the limitations of current bioinformatics’ methods for unequivocally identifying specific alleles from short-length reads (100–150 bp). To overcome such limitations, we developed a novel approach to define variables suitable for inferring gene abundance and gene diversity and, in our case, to perform quantitative analysis of antimicrobial resistance genes. Moreover, we suggest a workflow of variable normalization in relation to the information content of the targeted variable that would make it possible to compare different samples from various hosts. These tools were developed for ResCap but could be implemented for any other metagenomic sequence dataset. Shotgun metagenomic sequencing allowed assembling the sequences into contigs to infer the functionality of the sequenced metagenome. Figure 1 shows the workflow that illustrates and defines the variables used.

#### Raw data processing

Reads were mapped against our database, comprising ARG-ANNOT [27], BacMET [30], and ConjDB [33] databases independently, using Bowtie2 software [36]. Bowtie2 was set up to retrieve all end-to-end possible alignments and to suppress both discordant alignments and mixed alignments. The output SAM file was parsed to get the fields of Query template NAME, Reference sequence NAME, 1-based leftmost mapping Position, MAPping Quality, Position of the mate/next read*.* Reads with unavailable information (field Query Template NAME equal to ‘*’) were suppressed. Subsequently, a homemade Perl script was used to count matched reads per gene. Using the SAM parsed file and the length of the reference genes, the Perl script generated a table with the following fields: (i) the number of reads per gene mapped (“RPG”, gene depth coverage); (ii) the number of reads per kb of gene (“RPK”); (iii) the number of the reads that were mapped unequivocally to a given gene (“unique”); and (iv) the percentage of coverage of the gene sequence (“horizontal gene alignment coverage”) of each mapped gene. Table fields RPG, RPK, and unique, were normalized by the total amount of reads in each sample, transforming such fields in reads per gene per million reads and RPKM, respectively, the last being a common unit of gene abundance [37]. Several ways of normalizing abundance data have been applied to different studies (e.g., expression data in RNA-Seq experiments). The aim of our approach was to estimate the proportion of antimicrobial resistance genes among samples that putatively contained the same amount of DNA; thus, normalization using the total amount of DNA (i.e., reads) in the samples fits better with the initial approach.

**Fig. 2:**
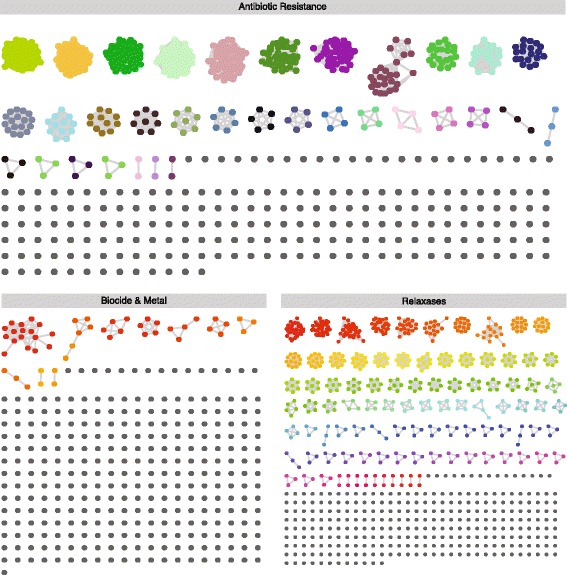
Allele network: nodes of the network represents individual genes that are mapped by some read. Edges between nodes represent reads that mapped on both nodes that link. Individual nodes are genes that are unequivocally identified. Gene clusters are mainly composed of different variants of the same gene (alleles). The Mapping Gene Cluster (MGC) is defined using the Markov cluster algorithm (MCL)

The redundancy of mapped reads can be represented as a network in which the nodes are the genes (usually alleles of the same gene), and the edges are the reads that map in the different nodes. Because one read can map in various alleles/genes, all the genes mapped by these reads are linked among them. The resulting network that comprises all the nodes and edges in a set of samples was named *allele network* (Fig. 2). The allele network must be unique for all samples of a given assay or study; thus, an allele network was built joining all the SAM parsed files of the study.

Each cluster of the allele network represents the set of alleles/genes detected by a set of reads and was defined as “mapping gene clusters” (MGCs). Each MGC can include hundreds of genes or just one gene, and will be detected when at least one read maps against any of the genes within that MGC. Due to allele network are constructed for the whole study, they constitute a set of normalized variables that allow performing qualitative and quantitative comparisons between the samples included in this particular analysis. To quantify the MGCs in each sample, the highest value shown by an allele (node) within a given MGC is the occurrence of such an MGC (abundance). This criterium is used to avoid an over and underestimation of data that would occur when using the mean or median of the reads corresponding to alleles with high homology (thus, sharing a high proportion of reads) but present in different proportions. Therefore, the MGC approach builds a unique variable for each set of possible detected alleles. Figure 2 shows the 839 MGCs of our sample (237 for AbR, 283 for biocides and metals and 319 for relaxases).

A homemade Perl script was used to build the allele network from the SAM parsed files, taking the mapped genes as nodes and searching the ambiguously mapped reads to create the edges. The Perl script calculates the edges’ weight as the number of reads that map the linked nodes at the same time. The allele network was loaded into the R environment [38] using the igraph package [39] MGCs were defined using *mcl,* from the MCL R package [40], with default parameters except allowing loops and clusters with only one member in the allele network.

##### Data analysis

The resistome of a given experiment was analyzed in terms of *gene abundance* and *gene diversity* according to the methodology described above. The abundance and the diversity of genes in a particular resistome are the (dependent) variables that define this resistome, which are measured as the number of RPKMs per MGC and the number of MGCs per million reads (MPM), respectively.

This MGC system builds a set of normalized variables that allow analyzing abundance and diversity within and between samples, and thus the comparison of datasets from various sources. The MGCs of the antibiotic resistance gene database were divided according to antibiotic families [27]. The MGCs of the relaxase database were organized in known different relaxase families [33, 41]. The MGCs of the biocide and heavy metal resistance gene database were classified according to susceptibility to specific compounds [30]. Biocide and heavy metals resistance genes that belong to more than one functional category (e.g., genes conferring resistance to various metals or genes encoding resistance to different biocides) contribute equally for any of them. Descriptive statistics were performed using the dplyr [42], tidyr [43], and ggplot2 [44] packages of R [38].

A statistical analysis of gene abundance, analogous to that used for comparing the abundance of mRNA among samples in differential expression analysis [37], was performed to quantify the relative abundance of the MGCs. Differential analysis was performed using the DESeq2 package [45]. Tables containing the original abundance data obtained by ResCap and MSS data sets were used as input for the DESeq2 package. Normalization and statistical analysis were performed with the default parameters of DESeq2. MGCs were classified as differentially detected (only present in either human or swine samples, *p* value ≤ 10^−3^) or commonly detected (present in both human and swine samples*, p* value ≥ 10^−3^) in different hosts. This *p* value ≤ 10^−3^ is also used as cutoff is other differential analysis.

#### Reference-free workflow

ResCap includes probes for approximately 78,600 genes that are homologous to known resistance genes, with different degrees of sequence identity, which might be involved in antimicrobial resistance. This ensemble of genes has also been considered in the definition of resistome [17]. Assemblies were performed by MegaHit software with default parameters [46]. Prodigal [47] was used for gene recognition and translation with the specific parameters for metagenomic sequences. Quality assemblies’ quantification was performed by Quast software [48]. Predicted genes were first annotated against the ResCap database by Best Blast Hit approach using blastn software [49]. To identify only the genes belonging to the ResCap database or their homologs and to minimize the false positive ratio, Blast hits were filtered by *e* value of 10^−100^ and 80% of reference coverage. Genes with identities higher than 95% and coverage higher than 80% were cataloged as belonging to ResCap database (ResCapDB). The remaining sequences were translated to proteins. Proteins were compared against UniProtKB database by blastp. Again, hits with higher identity than 95%, coverage higher than 80% and *e* value lower than 10^−100^ were considered as UniProtKB known proteins. The set of remaining proteins that did not accomplish this threshold were cataloged as novel proteins. Additional file 2 shows this analysis workflow.

### Samples analyzed

ResCap was validated by analyzing fecal samples from nine humans and eight swine, all collected as part of the FP7 European Research Consortium EvoTAR (http://www.evotar.eu). Independent fecal samples from swine were collected on Spanish farms linked to large companies that supply broilers and swine processed meat to the EU. Antibiotics as growth promoters or with preventive purposes are not used on these farms. Fecal human samples were collected at the Hospital Bichat, Paris, France, under the protocol approved by its local ethics committee. The DNA extraction from the fecal samples analyzed was performed according standardized protocols (MetaHIT Protocol; http://www.metahit.eu/). The robustness of the platform was tested by comparative analysis of two technical replicates of two swine samples.

## Results

ResCap performance was compared with MSS in two ways. First, by applying a reference-based approach that maps metagenome reads against specific databases (AbR, metals and biocides, and relaxases). Second, by applying a reference-free approach that assembles metagenomic reads and performs a functional annotation. The results of these evaluations appear below.

### Reference-based evaluation

This section addresses how the abundance and diversity of resistance genes (ResCap or those already validated) were calculated.

#### ResCap achieves better recovery of target genes than MSS

An average of 1.9 × 10^7^ paired reads was obtained from the MSS and ResCap datasets (0.92–3.2 × 10^7^). The on-target average (the number of reads mapping on the target genes relative to the total read number) against the selected databases (see “Methods”) was 0.11% (0.07–0.18) for MSS data and 30.26% (20.27–41.83%) for ResCap data, which represents an enrichment of 279-fold (Table 1).

**Table 1:** Summary of mapping results. Comparison of ResCap against MSS technology. The mapping ratios (on-target value) were extracted from the number of reads mapped (SAM files) divided by the total number of reads. The “gain values” are the results of dividing ResCap on-target value by the MSS on-target value

Sample	Metagenome shotgun sequence MSS					ResCap					Gain
	N° reads	AbR	BacMet	Rel	Total	N° reads	AbR	BacMet	Rel	Total	
Bichat1	14,127,290	0.05%	0.002%	0.04%	0.10%	16,705,789	19%	0.48%	5.22%	24.68%	244.24
Bichat2	15,128,135	0.05%	0.028%	0.04%	0.12%	33,589,838	12%	5.56%	2.98%	20.27%	170.81
Bichat3	14,488,245	0.05%	0.005%	0.03%	0.09%	17,276,637	34%	2.31%	5.96%	41.83%	480.59
Bichat6	17,476,666	0.07%	0.001%	0.05%	0.12%	19,191,320	25%	0.85%	5.84%	32.13%	261.87
Bichat7	16,732,926	0.07%	0.002%	0.05%	0.13%	28,530,922	27%	0.33%	5.99%	33.60%	267.77
Bichat9	17,058,000	0.03%	0.013%	0.05%	0.09%	18,038,257	14%	10.59%	7.55%	31.80%	336.29
Bichat10	15,039,883	0.03%	0.066%	0.06%	0.15%	34,798,281	6%	28.81%	5.48%	40.63%	265.17
Bichat11	13,425,077	0.03%	0.091%	0.06%	0.18%	35,901,508	5%	26.73%	4.72%	35.98%	201.67
Bichat13	17,903,872	0.05%	0.023%	0.06%	0.14%	26,283,052	16%	13.27%	7.39%	36.85%	270.02
F266	19,557,955	0.06%	0.005%	0.02%	0.08%	14,024,345	21%	4.62%	2.57%	28.23%	337.50
PIG20	27,375,311	0.08%	0.028%	0.01%	0.12%	22,485,364	18%	16.20%	1.81%	36.38%	298.23
PIG26	13,831,057	0.07%	0.005%	0.02%	0.10%	15,756,070	19%	3.88%	2.73%	25.93%	271.20
PIG29	18,945,765	0.09%	0.018%	0.02%	0.12%	26,223,850	18%	10.26%	2.52%	30.65%	248.31
PIG31	12,778,294	0.07%	0.010%	0.02%	0.09%	18,055,019	13%	5.76%	2.08%	20.77%	219.12
PIG528	19,689,471	0.06%	0.003%	0.02%	0.08%	13,864,257	21%	2.70%	3.11%	26.83%	323.23
PIG94	15,985,219	0.07%	0.004%	0.02%	0.10	15,351,408	18%	2.57%	3.31%	24.05%	240.20
PIG96	9,290,402	0.06%	0.001%	0.01%	0.07%	12,225,935	21%	1.13%	1.67%	23.84%	320.90

**Fig. 3:**
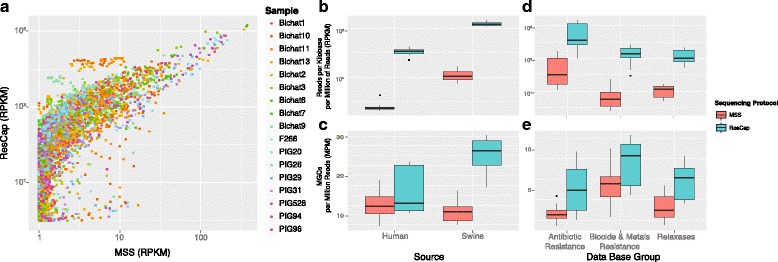
ResCap Performance Summary. Panel **a** represents the gain function in reads per kilobase per million of reads of each detected gene between MSS protocol (abscissa axis) and ResCap (ordinate axis). Genes only detected by ResCap are represented by the dot cluster in the initial values of the abscissa axis. Data distribution of the platform efficiency evaluating **b** the number of mapped reads per million of sequenced reads against a canonical (well known) gene data set; and **c** the number of detected genes per million of sequenced reads using as reference the well-known gene data set. Fecal samples were differentiated according to the source. Data distribution of the platform efficiency evaluating **d** the number of mapped reads per million of sequenced reads against the three canonical gene groups and **e** the number of detected genes per million of sequenced reads using as reference the three canonical gene groups

The analysis of gene abundance, expressed in reads per kb per million reads (RPKMs), demonstrates a better recovery of gene coding for resistance to antibiotics, heavy metals, biocides, and relaxases (plasmid genes), when using ResCap compared against MSS. Figure 3a represents the RPKMs inferred before (MSS) and after capture (ResCap) for all the samples analyzed, and Additional file 3 shows the gain plots for each sample. Most canonical genes (99.3%, 1.339/1.348) detected by MSS were also detected with ResCap.

Furthermore, a significant portion of genes detected by ResCap (42%, 975/2323), was not detected by MSS. The linearity of the system was evaluated by using a linear regression model for the genes only detected in each paired sample (MSS vs. ResCap). An *R*^2^ mean of 0.813 (0.85–0.99) shows a good match between both protocols.

The enrichment of canonical resistance genes when using ResCap was similar in samples from humans and swine. Further, the observed differences in the relative abundance of genes encoding resistance to antimicrobials (antibiotics, heavy metals, biocides) and relaxases in various samples (Fig. 3b, c) could be explained by the variability of microbiota from different hosts [50, 51].

#### ResCap addresses gene diversity

Allele redundancy of some resistance genes hinders the correct estimation of gene diversity and precludes a correct estimation of gene abundance in metagenomes when using most available metagenomic tools. To overcome this issue, we defined a mapping gene cluster (MGC) as a group of alleles/genes detected by the same set of reads and normalized such MGCs by the total number of sequencing reads per each sample expressed in millions of reads (MGC Per Million or MPM) in order to use it as unit of diversity that allow us comparing different samples. The number of MPMs increased 1.3-fold in humans (0.7–1.74, *p-value* 2·10^−1^) and 2.1-fold (2.3–1.9, *p-value* 2·10^−4^) in swine when using ResCap instead of MSS (Fig. 3b, c).

**Fig. 4:**
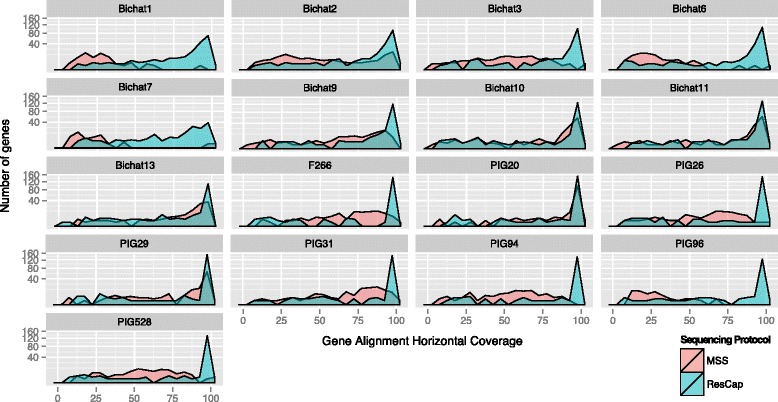
Longitudinal coverage distribution. The figure shows the comparison of longitudinal coverage distribution between protocols in each sample. Distributions are represented by density parameter and expressed by the number of genes (ordinate axis) and the coverage percent (abscissa axis)

**Fig. 5:**
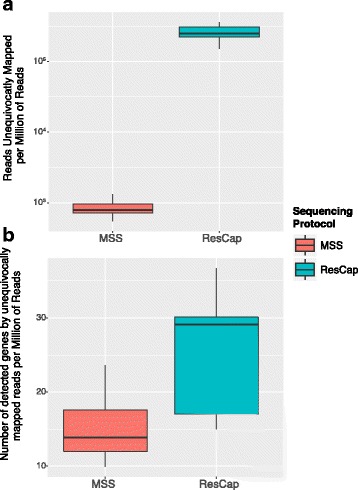
Quantification of unequivocally mapping reads. The figure shows the comparative of the quantification of reads mapping on just one gene (or allele). First, the abundance of reads that are unequivocally mapped on one gene (**a**). Second, the number of genes (or MGC) that have almost one read that maps unequivocally (**b**). Box plots are differentiated for MSS protocol and ResCap protocol

As an increase in reads per MGC does not imply a homogeneous distribution of the reads, we also determined the *gene horizontal alignment coverage*, which was defined as the fraction of a gene that is covered by reads, as well as the number of reads per nucleotide or *gene depth coverage*. These parameters determine the probability of identifying an allele-specific mutation by unequivocally mapping reads. Figure 4 shows how the horizontal gene alignment coverage obtained with MSS improves with ResCap from 73.4 to 97.5%, (35.9–94.8% vs. 66–99%). Most genes were almost fully covered by reads, with a general increase in gene depth coverage [Additional file 4]. Consequently, the number of genes unequivocally detected by ResCap was almost double that of MSS (*n* = 26, range 17.1–30.0 genes per sample per million of reads vs. *n* = 14.9, range 12–17.6 genes per sample per million of reads, respectively). The number of reads unequivocally mapped increased up to 300-fold (2 · 10^5^ for ResCap vs. 8 · 10^2^ for MSS) (Fig. 5).

**Fig. 6:**
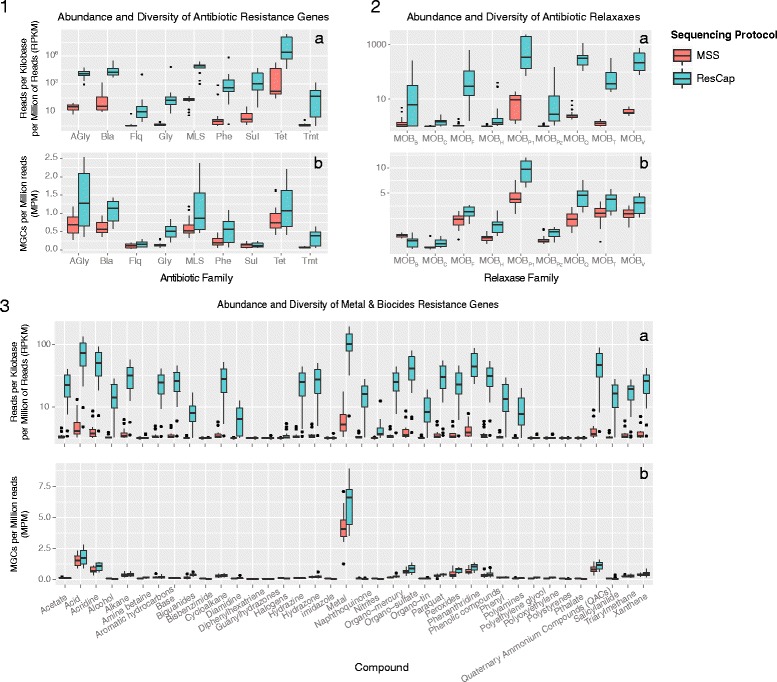
(1) Abundance and diversity of antibiotic resistances. Comparison of ResCap and MSS protocol in antibiotic resistance data. Antibiotic resistance genes were classified among nine antibiotic families (AGly: aminoglycosides, Bla: beta-Lactams, Flq: fluoroquinolones, Gly: glycopeptides, MLS: macrolides, Phe: phenicols, Sul: sulfonamides, Tet: tetracyclines and Tmt: trimethoprim). Abundance (**a**) was measured as read per kilobase per million reads that mapped against genes or allele-cluster genes of each antibiotic resistance family. Diversity (**b**) was measured as the number of detected genes per million reads of each antibiotic resistance family (2) Abundance and diversity of relaxases. Comparison of ResCap and MSS protocol in relaxases dataset. Relaxases were classified in nine protein families (MOB_B_, MOB_C_, MOB_F_, MOB_H_, MOB_P1_, MOB_P2_, MOB_Q_, MOB_T_, and MOB_V_). Abundance (**a**) was measured as read per kilobase per million reads that mapped against genes or allele-cluster genes of each relaxase family. Diversity (**b**) was measured as a number of detected genes per million reads of each relaxase family. (3) Abundance and diversity of biocide and metal resistances. Comparison of ResCap and MSS protocol in biocide and metal resistance data. Biocide and metal resistance genes were classified by the type of detoxified targets. Abundance (**a**) was measured as read per kilobase per million reads that mapped against genes or allele-cluster genes of each target family. Diversity (**b**) was measured as a number of detected genes per million reads of each target family.

Figure 6 shows the abundance (RPKMs) and diversity (MPMs) obtained by ResCap and MSS for individual categories of resistance genes (antibiotics, biocides and metals), which also illustrates the improved sensitivity of ResCap vs. MSS. Additional file 5 reflects that although both ResCap and MSS can track the most abundant gene families as those conferring resistance to beta-lactams, macrolides, aminoglycosides and tetracyclines followed by those conferring resistance to phenicols and sulfonamides, many canonical resistance genes were only detected by the ResCap platform (e.g., *mecA* and *blaZ* in beta-lactams; *ermA, ermC, ermD, erm33*, and *lnu* in macrolides; *fexA, catA*, and *catB* alleles in phenicols). Genes encoding resistance to fluoroquinolones, glycopeptides or trimethoprim, families of first-line antibiotics used to treat community and hospital-based infections, were barely detected using MSS but were unequivocally detected with ResCap (e.g., *dfrA16, dfrA15, dfrG*, and *dfrK* among those conferring resistance to trimethoprim; *oqxAB, qnrB*, and *qnrS* among those producing resistance to quinolones; and *vanB, vanA* for glycopeptide resistance). ResCap also detected more genes conferring resistance to heavy metals (e.g. cadmium, copper, silver and mercury) and relaxases, which are markers of plasmid families that carry antibiotic resistance genes (MOB_C_, MOB_F_, MOB_P1_, MOB_P2_)[Additional files 6, 7, and 8].

The robustness of the platform was ascertained by using replicates of swine samples. The correlation between replicates measured by Pearson’s linearity model test was 0.98 (*p* value 2.2 · 10^−16^) and 0.89 (*p* value 2.2 · 10^−16^), respectively [Additional file 9]. This result is within the range specified by NimbleGene for other SeqCapEZ capture platforms.

#### Comparative analysis of resistomes from various samples

**Fig. 7:**
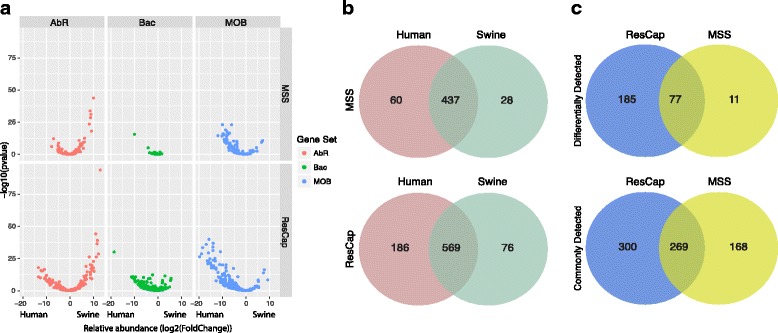
Differential study plots. Panel **a** shows the distribution of abundance variation between swine and human AbR resistomes (left), metal and biocide resistome (middle), and mobilome (right) in the form of volcano plots (fold change vs *p* value) using the different approaches MSS (top) and ResCap (bottom). Left and right branches in the volcano plot refers to higher abundance in humans and swine, respectively. Abscissa axis reflects the relative abundance between humans and swine samples. Positive values represent MGCs more abundant in swine than in human samples. Negative values represent MGCs more abundant in human than in swine samples and the values near to zero represent the MGCs with similar abundance between samples. Panel **b** summarizes the number of statistically significant MGCs of humans, swines, and the genes in common between them using both approaches: MSS (top) and ResCap (bottom). Panel **c** shows the Venn diagrams between approaches of differentially detected MGCs (top) and commonly (in both sets) detected MGCs (bottom)

In order to further demonstrate the improvements of using ResCap over MSS, we compared the resistomes of human and swine samples to estimate the relative abundance of MGCs by using these two methodological approaches. Volcano plots in Fig. 7a show a general view of the relative abundance of MGCs for each dataset (AbR, metal and biocide, and relaxases) and each method. MGCs for antimicrobials were more abundant in swine samples than humans while those for relaxases were predominant in human resistomes (represented as positive and negative *p* values, respectively, expressed in log_2_ in the *x*-axis). The figure also reflects the higher number of MGCs detected by ResCap than by MSS and the improvement of the *p* values.

Figure 7b represents the distribution of the MGCs in different hosts by using MSS and ResCap. Using MSS, the resistome of the total samples analyzed comprises 525 MGCs, 88 MGCs being differentially detected (60 MGCs from humans and 28 MGCs from swine), and 437 being commonly detected in these hosts. Conversely, ResCap detected 831 MGCS, 262 classified as differentially detected (186 from humans and 76 from swine) and 569 as commonly detected. The data reflects a 3-fold increase in the number of MGCs using ResCap in comparison with MSS.

Figure 7c compares the results obtained by ResCap and MSS for both the differentially detected and the commonly detected MGCs in different hosts (262 vs. 88, and 569 vs. 437, respectively).

Of all the MGCs differentially detected (262 by ResCap plus 88 by MSS), 185 were only differentially detected by ResCap, 77 were differentially detected by both approaches, and 11 were only differentially detected by MSS. The average of reads used for the statistical test of these 11 MGCs was 18 using MSS (ranging from 6.02 to 78.3 reads) and 2907 using ResCap (ranging from 1884 to 3947 reads), which make us to suggest these 11 MGCs could be potential false positives.

Of all MGCs commonly detected in both types of hosts (569 by ResCap and 437 by MSS), both methods detected 269 MGCs while 300 MGCs were only detected by ResCap and 168 MCGs were only detected by MSS. Again, the low number of useful reads might explain the higher *p* values of MSS over ResCap and identify this set of MGCs as common MGCs between humans and swine.

### Reference-free evaluation

**Fig. 8:**
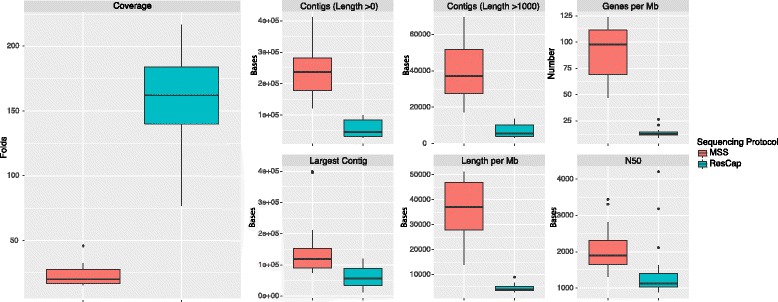
Assembly statistics. Assembly statistics was calculated by Quast software. Statistic summary of the main assembly variables; the number of contigs (all and longer than 1 kb), number of genes per sequenced Mb, the size of longest contig, the length of the assembled metagenome per sequenced Mb, and the N50 (the shortest contig length at 50% of the metagenome). Coverage data were calculated as the total sequenced bases divided by the total length (without normalizing). Length per Mb and genes per Mb were normalized by the total amount of megabases sequenced by each sample

Assembly statistics and coverage show that the information obtained with the ResCap platform only covers the small portion of the metagenome to which the platform has been designed (Fig. 8). The genes detected were classified in the ResCapDB, UniProtKB, and novel categories (see “Methods” for definition of each category).

**Fig. 9:**
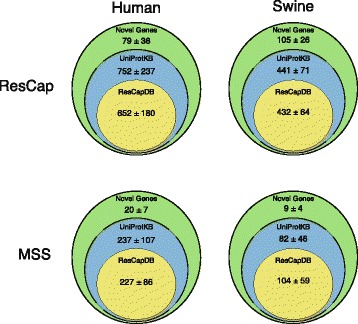
Functional annotation distribution. Assembled genes are classified as ResCapDB, UniProtKB, or novel genes (see “Methods”). All assessed genes have a maximum *e* value of 10^−100^ with some of the genes included in the ResCap design database. The figure shows the comparison between human and swine samples and between MSS and ResCap approaches

ResCap in comparison with MSS, improves not only the number of the detected canonical genes included in known databases (Arg-ANNOT [27], BACMet [30], and ConjDB [33]) as shown in previous sections, but also the number of detected ResCapDB, UniProtKB, or novel homologs as presented in Fig. 9 (ResCap_UniProtKB_ 752 ± 237 genes vs. MSS_UniProtKB_ 237 ± 107 for humans, and ResCap_UniProtKB_ 441 ± 71 genes vs. MSS_UniProtKB_ 82 ± 46 for swine; ResCap_Novel genes_, 79 ± 38 genes vs. MSS_Novel genes_ 20 ± 7107 for humans, and ResCap_Novel genes_ 105 ± 26 genes vs. MSS_Novel genes_ 9 ± 4 for swine)**.** In addition, Additional file 2 shows the better resolution of ResCap expressed by number of blast hits per gene per megabase. The actual role of these genes in antibiotic resistance will require functional validation that is beyond the scope of the current study. However, their identification as *bona fide* resistance genes as well as the analysis of changes in their abundance upon antibiotic challenge might have a significant impact on further studies on the evolution of antibiotic resistance.

## Discussion

This study reports the development of a novel targeted gene capture platform, ResCap, and its comparative evaluation with MSS in resistance gene identification using a collection of human and swine fecal samples. The results show that ResCap is suited for high-resolution analysis of resistomes and offers the possibility to detect genes homologous to known resistance genes, which will allow performing further comprehensive analyses on the diversity and the evolution of antibiotic resistance [17].

ResCap also provides several technical advantages to study resistomes in comparison with current metagenomic methods. First, the enrichment of ResCap resides in its targeted metagenomics approach, which significantly increases the recovery of resistance gene sequences. Our results indicate that the resistome represents barely 0.2% of the gut metagenome. As a consequence, MSS would need at least 3.75 · 10^9^ reads per sample to reach a similar coverage to that obtained by using ResCap (average of 1.9 · 10^7^ paired reads, which represents a relative enrichment of 279×). Second, the tiling of capture probes greatly facilitates the higher level of horizontal gene alignment coverage of ResCap as compared to MSS, resulting in increased specificity. Third, ResCap’s ability to detect previously unrecognized DNA fragments with homology to canonical resistance genes will facilitate the discovery of novel genes potentially involved in antimicrobial resistance. In case they were selectable, such novel genes would be enriched in the presence of antimicrobials, an important point to be tested during clinical trials. In addition, ResCap is of interest in public health, because it allows a more accurate risk ranking analysis [21] of the genes within the resistomes of various microbiota. Finally, the substantial capacity of the platform (200 Mb) makes ResCap extensible up to 2-fold of its current capacity, providing opportunities for updating the platform with probes for newly published resistance genes or for resistance genes added to resistance gene databases. ResCap updates will be publicly available through the GitHub repository (https://github.com/valflanza/ResCap) and the NimbleGene webpage. Nonetheless, the threshold of ResCap detection remains unknown due to the lack of a negative control that demonstrates the ability of ResCap to pick antibiotic resistance genes from quantified minority populations (e.g., mock genomic populations). Although appropriate, the complexity and variability of the metagenomic samples makes it difficult to use a good negative control for this type of study.

The parameters to express “gene abundance” and “gene diversity” allow comparing the resistomes of various samples. Relative abundance parameters are widely used in computational analysis of MSS datasets but require specialized statistics, because these compositional parameters are influenced by the variability in metagenomes of different samples. The novel “mapping gene cluster” (MGC) concept allows to provide a set of normalized variables that can be measured in terms of abundance and diversity among samples. Furthermore, MGC permits the evaluation of diversity within and among various functional groups (in our case, families of antibiotics, groups of genes conferring resistance to heavy metals or biocides and plasmid relaxases). To date, few quantitative metagenomic approaches to analyze resistomes are available, and they do not achieve this level of accuracy [14, 16].

Because of its sensitivity, specificity, and the possibility to compare results between samples, ResCap complies with the needs of public health epidemiology of antibiotic resistance that include (i) the detection of antibiotic resistance threats in various microbial environments [52, 53]; (ii) the need for implementation of accurate risk assessment studies based on resistome analysis in healthy humans, hospitalized patients, animal husbandry, the food industry, and the environment [52, 54]; (iii) the quality control of sewage and water body decontamination of antibiotic resistant genes [5, 54]; (iv) the update and refining of the list of resistance genes to be considered in monitoring the adverse effects of drugs in microbiomes, including pharmacomicrobiomic applications in clinical trials; (v) the close monitoring of the efficacy of microbiome reconstitution/rebiosis, whether through targeted probiotic live culture administration or fecal microbiota transplantation, to alleviate the adverse impact of antibiotic administration; and (vi) to analyze the effect of eco-evo drugs and strategies to combat antibiotic resistance [55].

## Conclusions

This study constitutes the first description of targeted metagenomics to analyze antimicrobial resistance. ResCap, the novel capture platform developed, allows meeting the challenge of analyzing samples with a complex and heterogeneous mix of genes in low and high concentration DNA samples with a high level of specificity and to further explore the presence of novel genes. Thus, ResCap-like approaches might also be used to identify other sequences in minority bacterial populations that are part of complex microbial systems, such as virulence determinants, key ecological traits involved in biosynthesis or biodegradation, or relevant genes of biotechnological interest.

## Additional files

Additional file 1: Histogram of gene coverage distribution by hybridizing probes. Two metrics were provided by NimbleGene: direct coverage (red bars) and adjacent coverage (cyan bars). Ninety percent of the genes are covered by at least 96.9% of direct coverage, and 90% of the genes are covered by 100% of adjacent coverage. (PDF 173 kb)

Additional file 2: Blast annotations summary. Summary of the classification steps of assembled genes. The sequential annotation comprises a first blastn search for identifying homologous resistome genes. Genes with an e-value higher than 10^−100^ were discarded. Filtered genes were split into two groups: genes with identity higher than 95% and genes with identity lower than 95%. The second group was annotated against UniProtKB and was split again into two groups: genes with identity higher than 95% and genes with identity lower than 95%. A number of blast hits were normalized by the number of assembling genes per sequenced megabase. (PDF 351 kb)

Additional file 3: Gain function plot for each sample. Representation of the gain in reads per kilobase per million reads of each detected gene between MSS (abscissa axis) and ResCap (ordinate axis). Genes, which were only identified by ResCap, are represented by the dot cluster in the initial values of the abscissa axis. The pictures are represented in log-log scale to better perceive the linearity of the gain function in genes detected by each protocol. (PDF 1129 kb)

Additional file 4: Distribution of read abundance. Figure shows the histograms of read abundance per each gene. Each frame represents a sample, superimposing results from the MSS protocol and the ResCap protocol. A square scale was used for the ordinate axis and a logarithmic scale for the abscissa axis to optimize the representation of the data. (PDF 178 kb)

Additional file 5: MGC abundance comparison of antibiotic resistance between swine and human samples. MGCs corresponding to the antibiotic resistance dataset were classified by antibiotic families (Agly: aminoglycosides, Bla: betalactams, Flq: fluoroquinolones, Gly: glycopeptides, MLS: macrolides, Phe: phenicols, Sul: sulfonamides, Tet: tetracyclines, Tmt: trimethoprim). Abundance was measured as read per kilobase per million reads. The right panel shows the results of MSS, and the left panel shows the results of ResCap. (PDF 533 kb)

Additional file 6: MGC abundance comparison of biocide resistance between swine and human samples. Gene abundance was extracted from original count data after normalization. Some sets of genes make complex MGCs. In this representation, MGC quantification was discarded in order to increase the biological information. Genes were classified by compound susceptibility. Because some biocide resistance genes can confer different phenotypes (resistance to more than one compound), genes are not constricted to one category. Genetic abundance is expressed as reads per kilobase per million reads (RPKM). The right panel shows the results of MSS and the left panel shows the results of ResCap. (PDF 933 kb)

Additional file 7: Gene abundance comparison of metal resistance between swine and human samples. Gene abundance was extracted from original count data after normalization. Some sets of genes make complex MGCs. In this representation, MGC quantification was discarded in order to increase the biological information. Genes were classified by metal susceptibility. Because some metal resistance genes can confer different phenotypes (resistance to more than one compound), genes are not constricted to one category. Genetic abundance is expressed as reads per kilobase per million reads (RPKM). The right panel shows the results of MSS and the left panel shows the results of ResCap. (PDF 602 kb)

Additional file 8: MGC abundance comparison of relaxases between swine and human samples. Relaxases were classified by MOB families. MGC abundance was summarized in MOB families. Each MOB family is composed of several MGCs. Genetic abundance is expressed as reads per kilobase per million reads (RPKM). The right panel shows the results of MSS, and the left panel shows the results of ResCap. (PDF 116 kb)

Additional file 9: Reproducibility of ResCap. Reads from replicates are represented in dot plot to illustrate the linearity of the results from ResCap sequencing. Dots represent the genes detected in any of the replicates. Pearson’s product-moment correlation was used to estimate the correlation between technical replicates. (PDF 295 kb)
