# Supplementary figures and images for: In-depth resistome analysis by targeted metagenomics
Source: Microbiome. 2018 Jan 15;6:11. doi: 10.1186/s40168-017-0387-y (PMC5769438; doi:10.1186/s40168-017-0387-y)

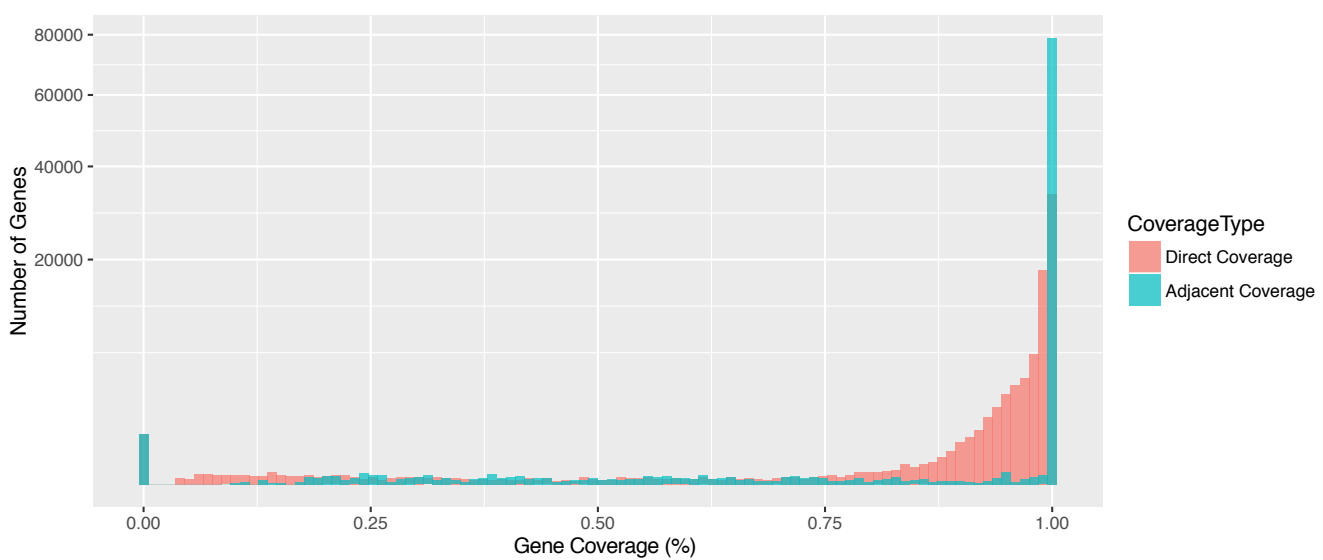

Supplement: Supplementary file 1 — Histogram of gene coverage distribution by hybridizing probes. Two metrics were provided by NimbleGene: direct coverage (red bars) and adjacent coverage (cyan bars). Ninety percent of the genes are covered by at least 96.9% of direct coverage, and 90% of the genes are covered by 100% of adjacent coverage. (PDF 173 kb) [file 40168_2017_387_MOESM1_ESM.pdf]

Number of Blast Hits per Genes per Megabase

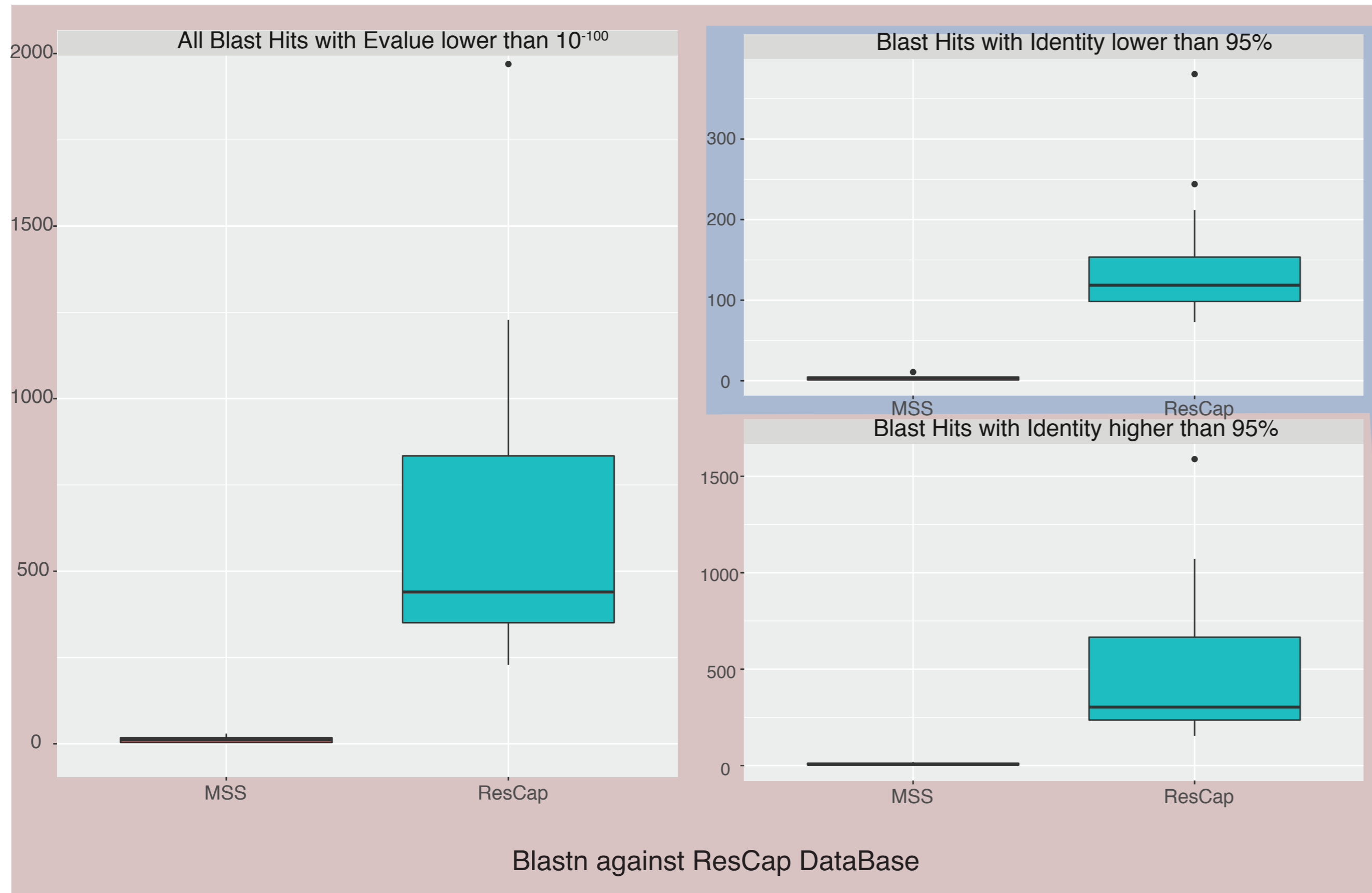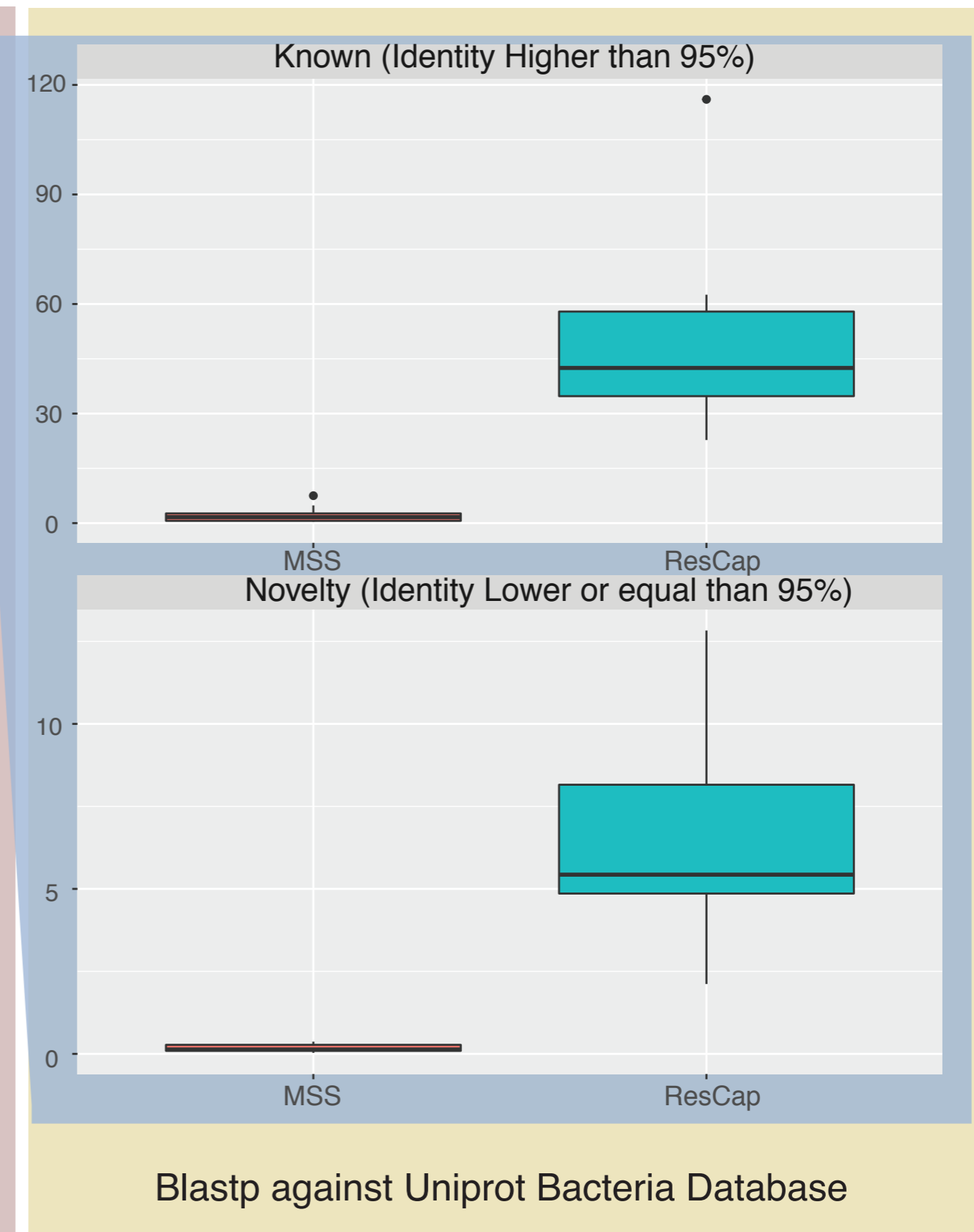

Sequencing Protocol

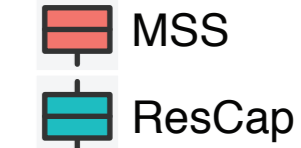

Supplement: Supplementary file 2 — Blast annotations summary. Summary of the classification steps of assembled genes. The sequential annotation comprises a first blastn search for identifying homologous resistome genes. Genes with an e-value higher than 10−100 were discarded. Filtered genes were split into two groups: genes with identity higher than 95% and genes with identity lower than 95%. The second group was annotated against UniProtKB and was split again into two groups: genes with identity higher than 95% and genes with identity lower than 95%. A number of blast hits were normalized by the number of assembling genes per sequenced megabase. (PDF 351 kb) [file 40168_2017_387_MOESM2_ESM.pdf]

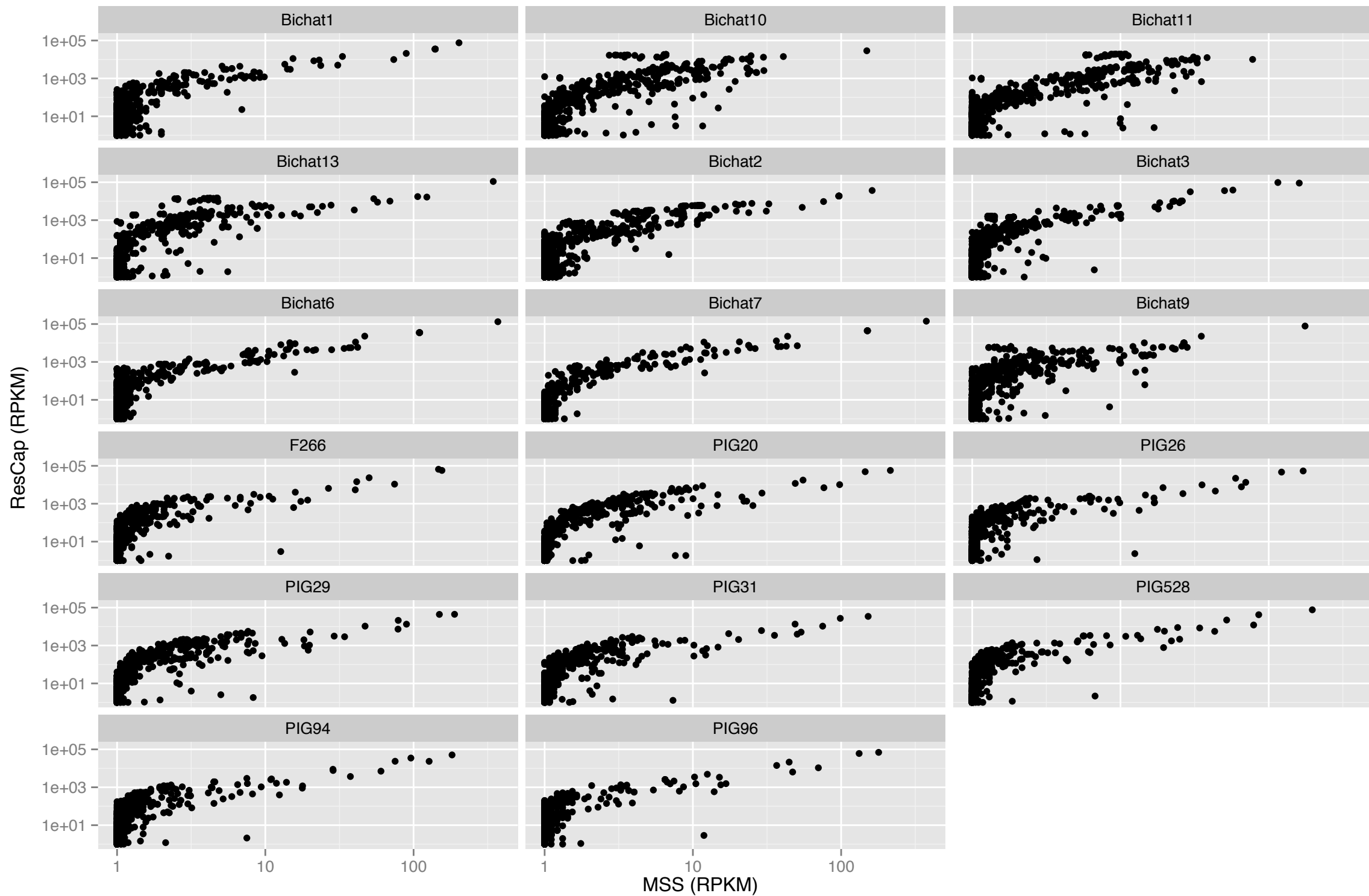

Supplement: Supplementary file 3 — Gain function plot for each sample. Representation of the gain in reads per kilobase per million reads of each detected gene between MSS (abscissa axis) and ResCap (ordinate axis). Genes, which were only identified by ResCap, are represented by the dot cluster in the initial values of the abscissa axis. The pictures are represented in log-log scale to better perceive the linearity of the gain function in genes detected by each protocol. (PDF 1129 kb) [file 40168_2017_387_MOESM3_ESM.pdf]

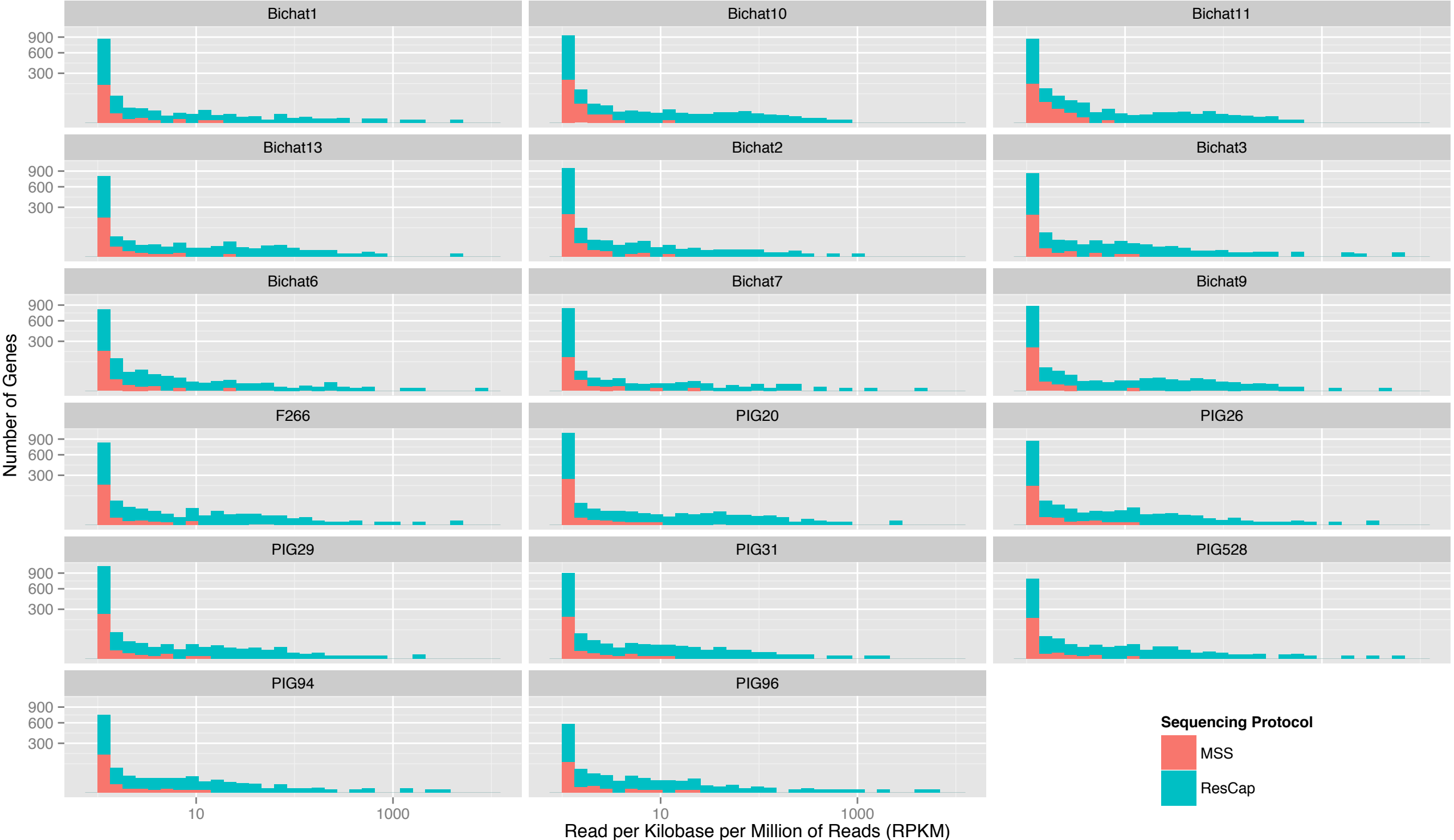

Supplement: Supplementary file 4 — Distribution of read abundance. Figure shows the histograms of read abundance per each gene. Each frame represents a sample, superimposing results from the MSS protocol and the ResCap protocol. A square scale was used for the ordinate axis and a logarithmic scale for the abscissa axis to optimize the representation of the data. (PDF 178 kb) [file 40168_2017_387_MOESM4_ESM.pdf]

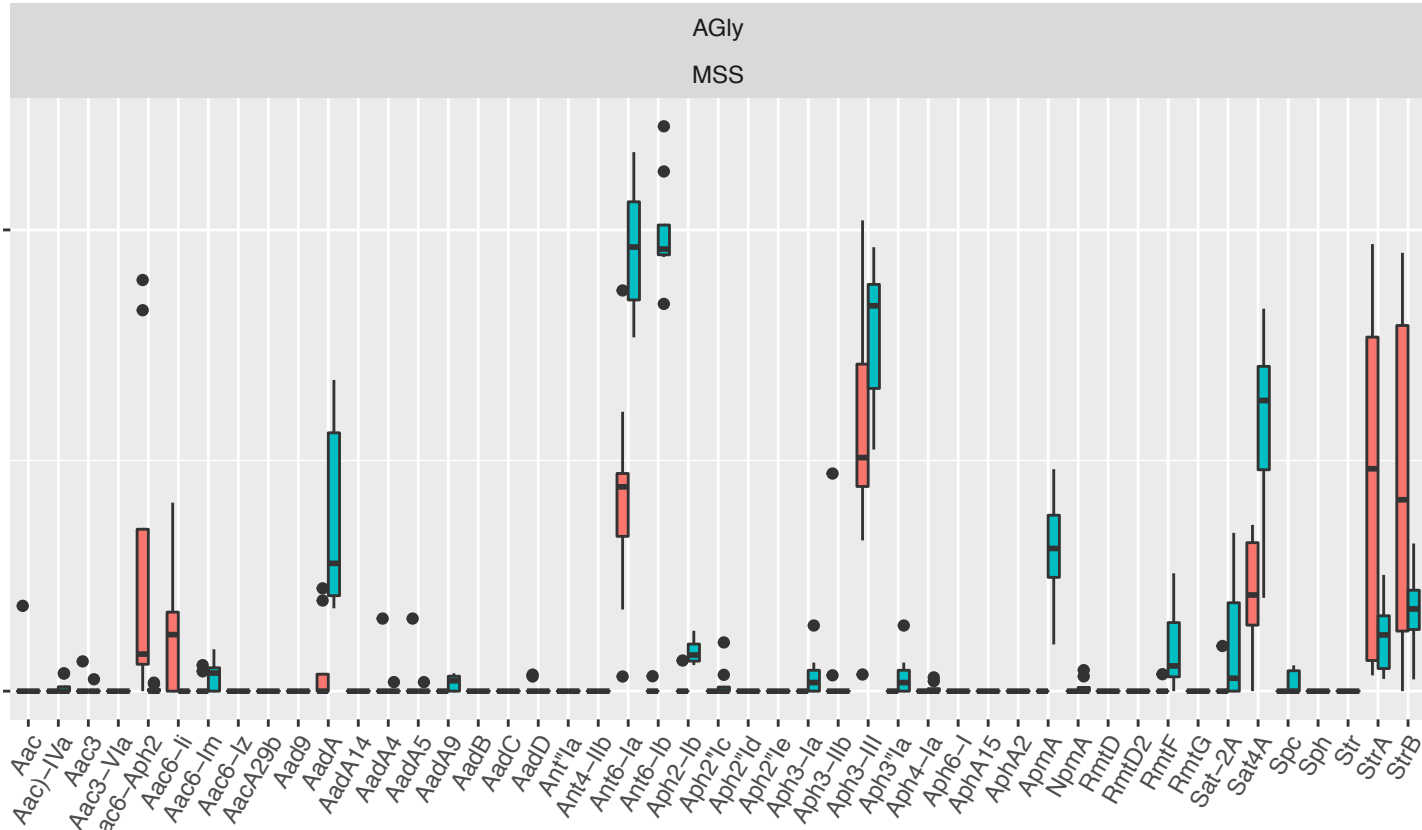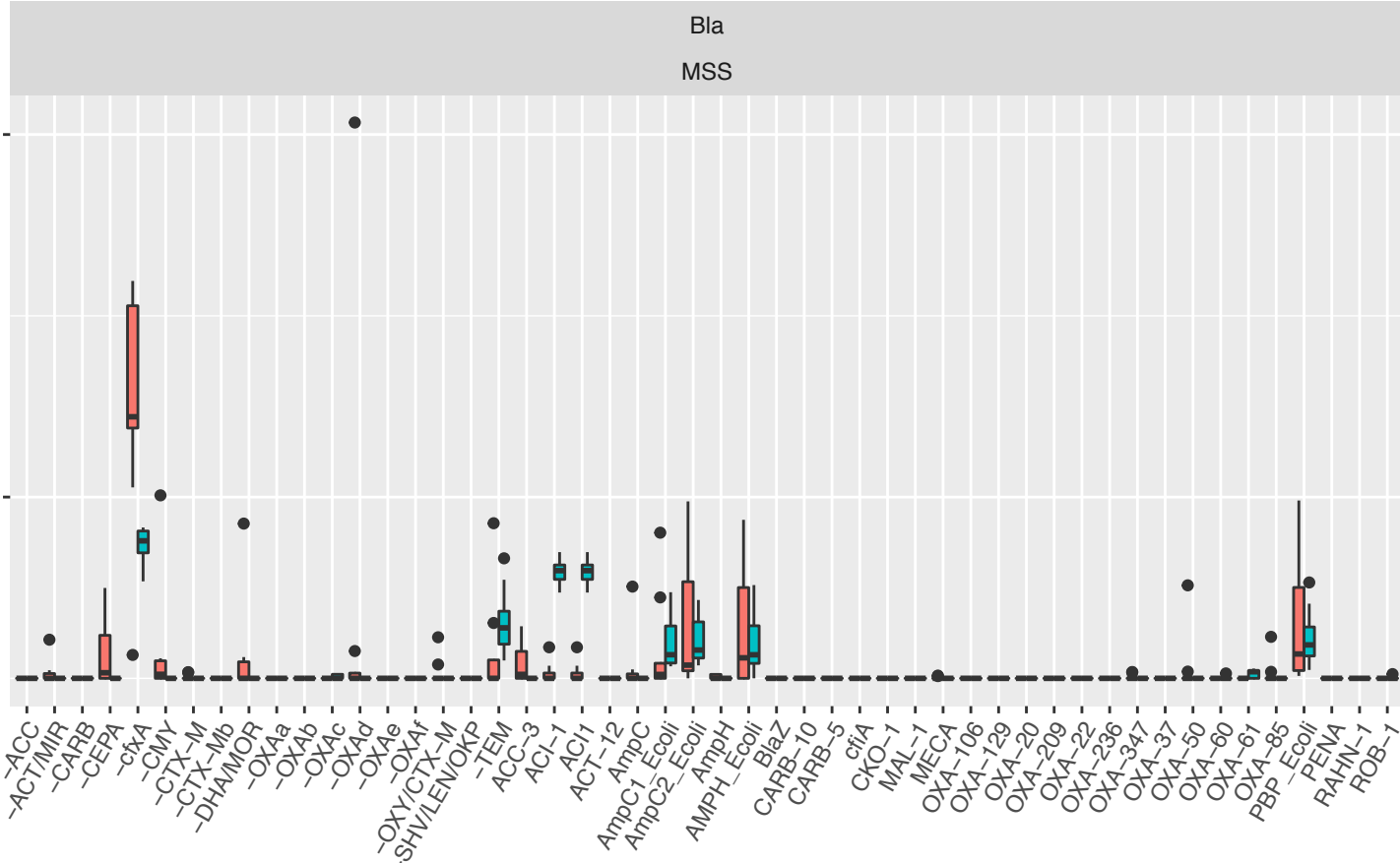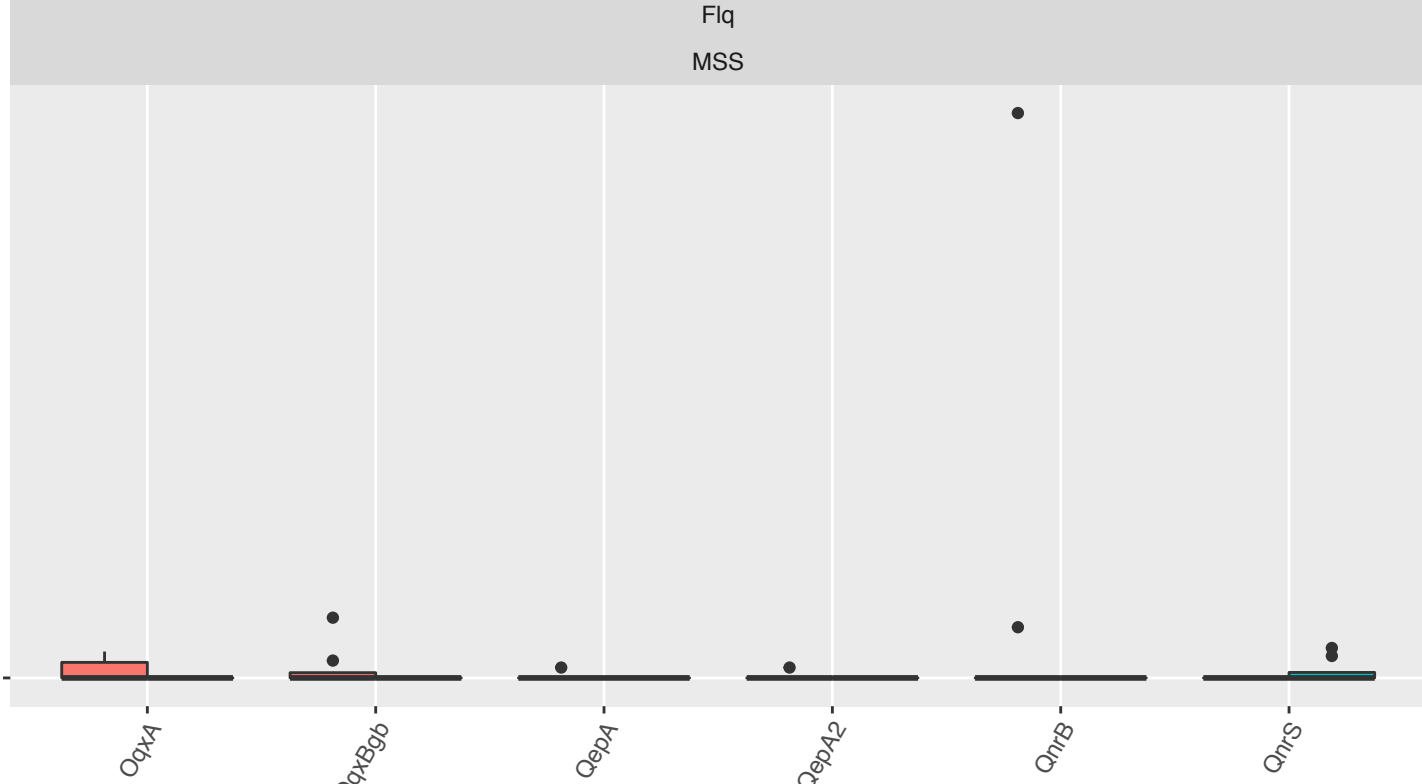

Supplement: Supplementary file 5 — MGC abundance comparison of antibiotic resistance between swine and human samples. MGCs corresponding to the antibiotic resistance dataset were classified by antibiotic families (Agly: aminoglycosides, Bla: betalactams, Flq: fluoroquinolones, Gly: glycopeptides, MLS: macrolides, Phe: phenicols, Sul: sulfonamides, Tet: tetracyclines, Tmt: trimethoprim). Abundance was measured as read per kilobase per million reads. The right panel shows the results of MSS, and the left panel shows the results of ResCap. (PDF 533 kb) [file 40168_2017_387_MOESM5_ESM.pdf]

Reads per Kilobase per Million of Reads (RPKM)

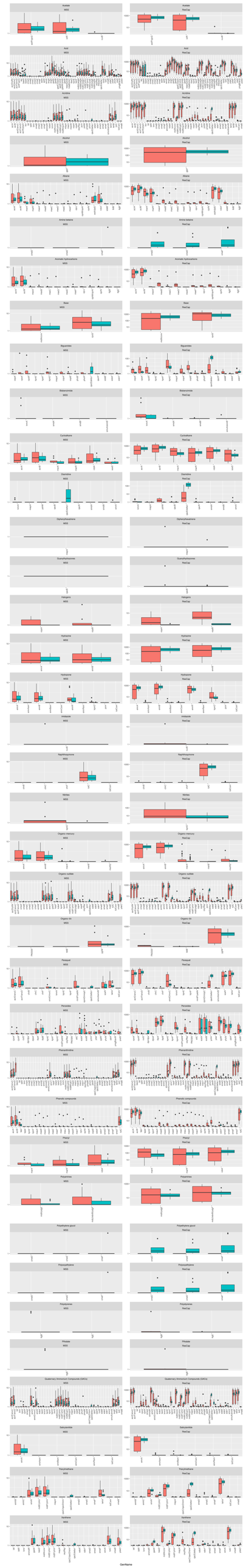

Supplement: Supplementary file 6 — MGC abundance comparison of biocide resistance between swine and human samples. Gene abundance was extracted from original count data after normalization. Some sets of genes make complex MGCs. In this representation, MGC quantification was discarded in order to increase the biological information. Genes were classified by compound susceptibility. Because some biocide resistance genes can confer different phenotypes (resistance to more than one compound), genes are not constricted to one category. Genetic abundance is expressed as reads per kilobase per million reads (RPKM). The right panel shows the results of MSS and the left panel shows the results of ResCap. (PDF 933 kb) [file 40168_2017_387_MOESM6_ESM.pdf]

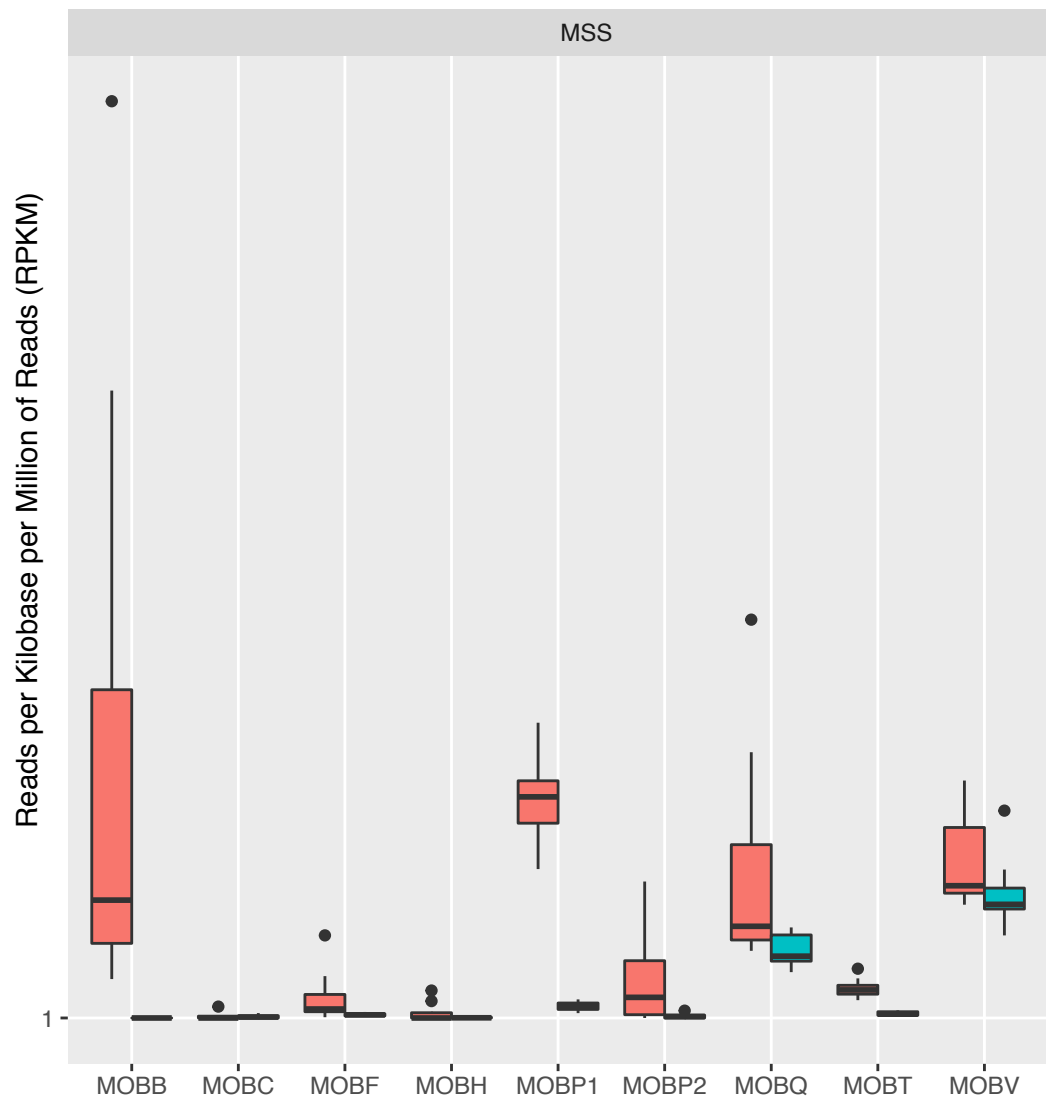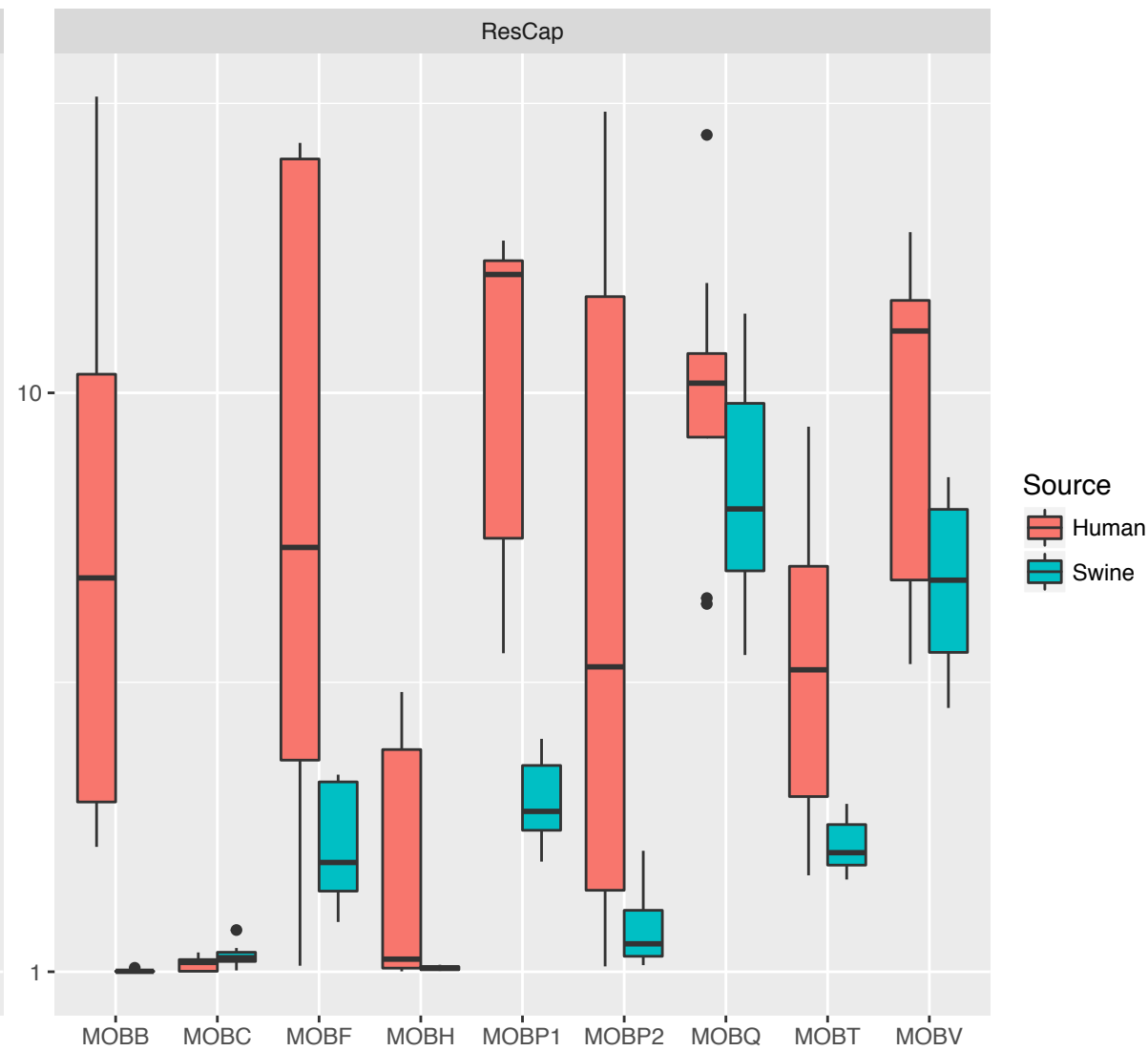

Supplement: Supplementary file 8 — MGC abundance comparison of relaxases between swine and human samples. Relaxases were classified by MOB families. MGC abundance was summarized in MOB families. Each MOB family is composed of several MGCs. Genetic abundance is expressed as reads per kilobase per million reads (RPKM). The right panel shows the results of MSS, and the left panel shows the results of ResCap. (PDF 116 kb) [file 40168_2017_387_MOESM8_ESM.pdf]

Number of reads in Replicate 1

PIG28

$R^2 = 0.977$

PIG94

$R^2 = 0.897$

Sample

PIG28

PIG94

Number of reads in Replicate 2

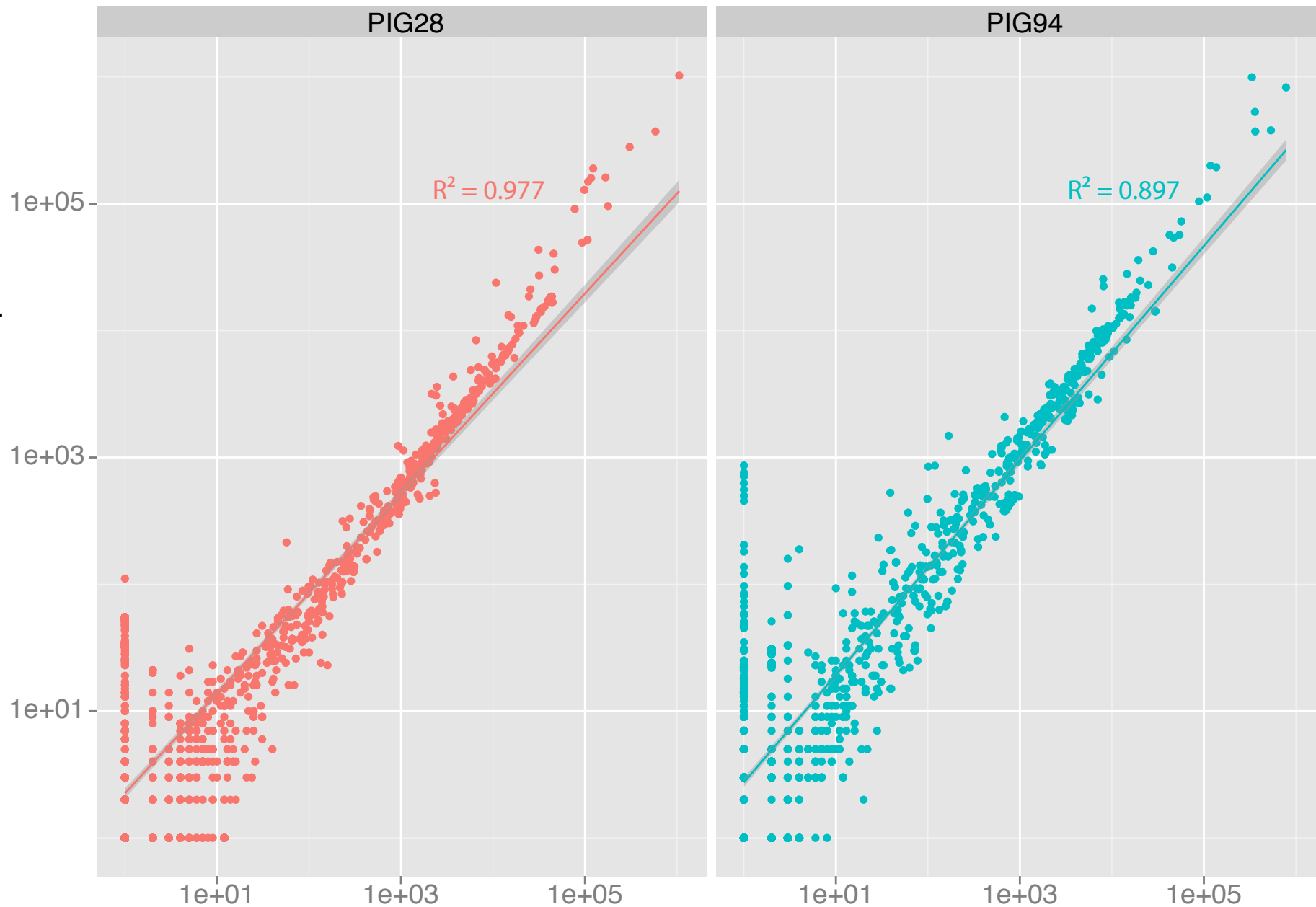

Supplement: Supplementary file 9 — Reproducibility of ResCap. Reads from replicates are represented in dot plot to illustrate the linearity of the results from ResCap sequencing. Dots represent the genes detected in any of the replicates. Pearson’s product-moment correlation was used to estimate the correlation between technical replicates. (PDF 295 kb) [file 40168_2017_387_MOESM9_ESM.pdf]
