# Supplementary material for: In-depth resistome analysis by targeted metagenomics
Source: Microbiome. 2018 Jan 15;6:11. doi: 10.1186/s40168-017-0387-y (PMC5769438; doi:10.1186/s40168-017-0387-y)
Supplement: Supplementary file 7 — Gene abundance comparison of metal resistance between swine and human samples. Gene abundance was extracted from original count data after normalization. Some sets of genes make complex MGCs. In this representation, MGC quantification was discarded in order to increase the biological information. Genes were classified by metal susceptibility. Because some metal resistance genes can confer different phenotypes (resistance to more than one compound), genes are not constricted to one category. Genetic abundance is expressed as reads per kilobase per million reads (RPKM). The right panel shows the results of MSS and the left panel shows the results of ResCap. (PDF 602 kb) [file 40168_2017_387_MOESM7_ESM.pdf]

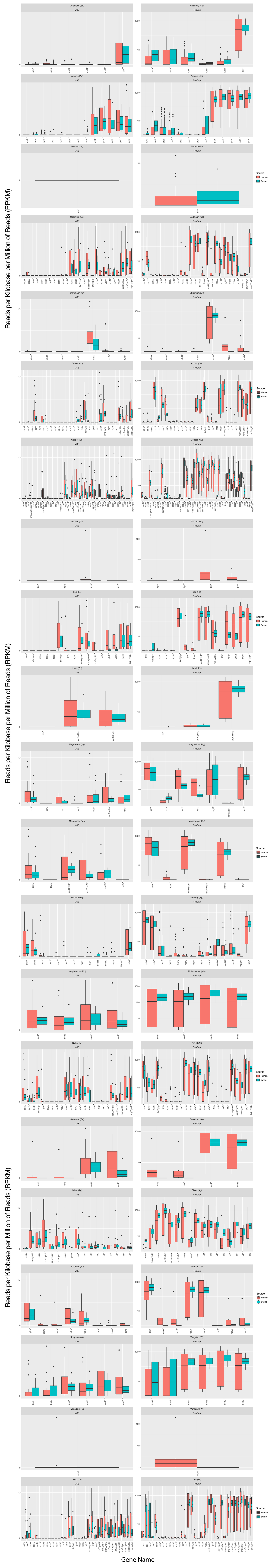
Reads per Kilobase per Million of Reads (RPKM)
Reads per Kilobase per Million of Reads (RPKM)
Reads per Kilobase per Million of Reads (RPKM)
Source
Human
Swine
Gene Name
